# *Pseudomonas aeruginosa*-derived metabolites and volatile organic compounds: impact on lung epithelial homeostasis and mucosal immune response

**DOI:** 10.3389/fimmu.2025.1553013

**Published:** 2025-06-09

**Authors:** Shanny Hsuan Kuo, Gee W. Lau

**Affiliations:** Department of Pathobiology, College of Veterinary Medicine, University of Illinois Urbana-Champaign, Urbana, IL, United States

**Keywords:** *Pseudomonas aeruginosa*, volatile organic compounds, bacterial metabolites, airway mucus dysregulation, pulmonary immunity

## Abstract

Pulmonary diseases, such as cystic fibrosis (CF), chronic obstructive pulmonary disease (COPD), and ventilator-associated pneumonia (VAP), are attributed to the prolonged infection of the airway and hypersecretion of mucus. *Pseudomonas aeruginosa* (PA) is one of the most common nosocomial pathogens in these diseased airways, secreting a wide spectrum of metabolites and volatile organic compounds (VOCs) that significantly impact the respiratory epithelium, including disruption of mucus homeostasis and inflammatory responses of the diseased lungs. In this review, we highlighted the major metabolites and VOCs produced by PA and the mechanisms by which they modulate inflammation, cellular injury, and mucus hypersecretion in respiratory epithelium.

## 1 Introduction

*Pseudomonas aeruginosa* (PA) is a common Gram-negative opportunistic bacterial pathogen that colonizes the respiratory tracts of individuals suffering from chronic lung diseases such as cystic fibrosis (CF), advanced stages of chronic obstructive pulmonary disease (COPD), bronchiectasis, and chronic bronchitis (CB); as well as ventilator-associated pneumonia (VAP) (1). Infections are most commonly associated with increased morbidity, pulmonary function deterioration, and prolonged hospitalization (2, 3). PA is extremely versatile metabolically, and is capable of producing a plethora of virulence factors, volatile organic compounds (VOCs), and secondary metabolites, which contribute to its pathogenicity in mammalian hosts, environmental adaptability, and interactions with other microorganisms. The persistent presence of PA is often linked to poor clinical outcomes. In this review, we describe the effects of various PA secondary metabolites and VOCs on respiratory epithelial cells and local lung inflammation. In particular, it examines how these metabolites participate in mucus imbalance, epithelial injury, and pneumonic inflammation, providing clues about the disease pathogenesis during PA infections.

## 2 *Pseudomonas aeruginosa*-derived volatile organic compounds

As is the case with all living creatures, bacteria emit a wide variety of VOCs. Some of these VOCs are unique to specific bacterial species and are useful biomarkers for pathogen identification. These VOCs encompass a diverse range of metabolites generated through microbial growth, serving as indicators of cellular signaling and metabolic activities (4). PA emits a list of unique VOCs during lung infections that are identifiable through recently improved detection methodologies, and have sparked growing interest in associating the presence of specific VOC profiles for clinical applications (5–7), aiming to improve diagnostic accuracy for disease detection and monitoring. The swift and precise identification of the causative pathogen is essential for the effective administration of targeted, narrow-spectrum antimicrobial treatment. Additionally, early diagnosis of PA infection, combined with appropriate antibiotic treatment, may facilitate the eradication of the pathogen before the infection progresses to a chronic state. However, current diagnostic methods are primarily based on the microbiological culture of respiratory specimens (8). This approach is often protracted, typically requiring three days, invasive, and not routinely performed following initial clinical suspicion of PA infection (9, 10). In fact, both the detection and monitoring of PA lung infections traditionally rely on sputum cultures. However, with advancements in highly effective modulator therapy in CF, sputum production has decreased, even though the risk of lung infections remains. A promising alternative to address these limitations is to monitor shifts in the molecular phenotype of either the host or the bacterial metabolism by analyzing distinct VOC profiles (11). Consequently, many laboratories have attempted to identify such biomarkers by analyzing VOCs released from *in vitro* PA cultures and in patients, as detailed in **Tables 1**, **2**. These investigations are largely based on gas chromatography-mass spectrometry (GC-MS), frequently coupled with solid-phase microextraction (SPME), and on selected ion flow tube mass spectrometry (SIFT-MS) and proton transfer reaction mass spectrometry (PTR-MS) (6, 7). Notably, the assessment of VOCs from respiratory samples in human subjects, such as bronchoalveolar lavage fluid (BALF), sputum, sinus mucus, and exhaled breath, has been suggested as a minimally invasive method for diagnosis and monitoring of PA lung and sinus infections, particularly in conditions such as CF (12–22).

**Table 1:** Summary of volatile organic compounds (VOCs) detected in *in vitro* studies involving *P. aeruginosa*.

*In vitro*	Volatiles detected^*^	References
Bacterial culture	acetaldehyde, acetic acid, acetone, ammonia, ethanol, dihydrogen sulfide, dimethyl disulfide, dimethyl sulfide, methyl mercaptan	Allardyce et al. (2006) (39)
Bacterial culture	3-methyl-1-butanol, ethanol, 2-butanol, **2-nonanone**, 2-pentanone, 2-heptanone, 4-heptanone, 3-octanone, 2-butanone, methyl isobutyl ketone, ethyl acetate, methyl 2-methylbutyrate, methyl methacrylate, ethyl 2-methylbutyrate, 2-methylbutyl isobutyrate, isoamyl butyrate, 2-methylbutyl 2-methylbutyrate, amyl isovalerate, dimethyl sulfide, dimethyl disulfide, dimethyl trisulfide, methanethiol, mercaptoacetone, 2-methoxy-5-methylthiophene, 3-(ethylthio)-propanal, **1-undecene**, 2-methyl-2-butene, **1,10-undecadiene**, **1-nonene**, **1-decene**, **1-dodecene**, butane, isoprene, 10-methyl-1-undecene, pyrrole, **3-methylpyrrole**, **1-vinyl aziridine**	Filipiak et al. (2012) (35)
Bacterial culture	**hydrogen cyanide,** ammonia, methyl mercaptan	Carroll et al. (2005) (25)
Bacterial culture	hydrogen cyanide	Gilchrist et al. (2011) (26)
Bacterial culture	2-aminoacetophenone, ammonia, ethanol, formaldehyde, hydrogen sulfide, isoprene, methyl mercaptan, trimethylamine	Thorn et al. (2011) (28)
Bacterial culture	2-aminoacetophenone, 2-pentanone, 4-methylphenol, acetic acid, acetone, acetonitrile, ethanol, ethylene glycol, indole	Zhu et al. (2010) (27)
Bacterial culture	1-butanol, 1-undecene, 2-butanone, 2-heptanone, 2-nonanone, 2-undecanone, 3-methyl-1-butanol, toluene	Zechman et al. (1985) (34)
Bacterial culture	2-aminoacetophenone	Cox et al. (1979) (29)
Bacterial culture	1-undecene, 2-aminoacetophenone, 2-butanone, 2-nonanone, 2-undecanone, 3-methyl-1-butanol, 4-methyl-quinazoline, butanol, dimethyl disulfide, dimethyl trisulfide, methyl mercaptan, toluene	Labows et al. (1980) (30)
Bacterial culture	2-propanol	Wang et al. (2006) (19)
Bacterial culture	2-aminoacetophenone, dimethyl disulfide, dimethylpyrazine, dimethyl sulfide, undecene	Preti et al. (2009) (22)
Bacterial culture	Methyl thiocyanate	Shestivska et al. (2011) (18)
Bacterial culture	ethanol, acetone, 2-butanone, 2-pentanone, isoprene, 2-aminoacetophenone, dimethyl sulphide, dimethyl disulphide, dimethyl trisulphide, methyl thiocyanate, 3-methyl-butanone, acetophenone, methylthioacetate and methyl thiobutanoate, hydrogen cyanide	Shestivska et al. (2012) (31)
Bacterial culture	acetone, dimethyl sulfide, dimethyl disulfide, dimethyl trisulfide, methyl isobutyl ketone, 3-methyl-2-pentanone, methyl vinyl ketone, 2-butanone, 3-methyl-2-butanone, 3-methyl-3-buten-2-one, 2-pentanone, 2,4-dimethylfuran, 2,3-pentanedione, 2,3-hexanedione, pyrrole, 2,4-dimethyl-1-heptene, 3-methyl-3-penten-2-one, 3-heptanone, 2-heptanone, decane, 3-octanone, 2,4,6-trimethylpyridine, 2-nonanone, acetophenone, 2-decanone	Bean et al. (2016) (37)
Bacterial culture	3-methylbutanal, thiocyanic acid, methyl ester, 3-methyl-1-butanol, pyridine, 3-penten-2-one, 3-methyl-1-buten-1-ol, 4-methyl-3-penten-2-one, hexanal, heptanal, 2,5-dimethylpyrazine, 2-nonanone, 4-ethyl-1,2-dimethylbenzene, 1,3,3-trimethyl-(bicyclo)-heptan-2-ol, 1-(4-ethylphenyl)-ethanone	Davis et al. (2020) (36)
Bacterial culture	1-undecene, methyl thiocyanate, dimethyl sulfide, 2-aminoacetophenone	Ahmed et al. (2023) (32)
Cell culture (A549 epithelial cells co-culture)	3-methyl-1-butanol, acetone, ethanol, ethylidenecyclopropane, tert-butyl ethyl ether, methyl tert butyl ether	Oluwasola et al. (2018) (38)

^*^The volatile molecules highlighted in bold are specifically suggested as potential biomarkers for *P. aeruginosa* infection among all the VOCs listed in that study.

**Table 2:** Literature overview of volatile organic compounds (VOCs) detected in *in vivo* studies involving *P. aeruginosa*.

*In vivo* (human)	Volatiles detected^*^	Diseases associated	References
Breath	**hydrogen cyanide**	cystic fibrosis	Enderby et al. (2009) (16) Gilchrist et al. (2013) (17) Smith et al. (2013) (23)
Breath	methyl thiocyanate	cystic fibrosis	Shestivska et al. (2011) (18)
Breath	**2-aminoacetophenone**	cystic fibrosis	Scott-Thomas et al. (2010) (13)
Breath	2-propanol	cystic fibrosis	Wang et al. (2006) (19)
Sinus mucus	2-aminoacetophenone, 2-methylbutyric acid, 3-hydroxy-2-butanone, acetamide, acetic acid, acetone, dimethyl disulfide, dimethyl sulfide, dimethyl sulfone, hydrogen sulfide, indole, isovaleric, phenol, propanoic acid	sinusitis	Preti et al. (2009) (22)
Sputum	1-heptene, **2-nonanone**, 2,4-dimethyl-heptene, 3-methyl-1-butanol, limonene	bronchiectasis and cystic fibrosis	Savelev et al. (2011) (14)
Sputum	1-undecene, 1-α-pinene, dodecane, terpinen-4-ol, 2,2,6-trimethyl-octane	bronchiectasis	Goeminne et al. (2012) (20)
Sputum	hydrogen cyanide	cystic fibrosis and non-cystic fibrosis bronchiectasis	Ryall et al. (2008) (24)
Bronchoalveolar lavage	2-butanone, 3-methyl-2-butanone	cystic fibrosis	Nasir et al. (2018) (21)

^*^The volatile molecules highlighted in bold are specifically suggested as a potential biomarker for *Pseudomonas aeruginosa* infection in that study.

### 2.1 PA-derived VOCs detected *in vitro* and *in vivo*

Key VOCs identified for PA include hydrogen cyanide (HCN), a well-known compound that has been consistently detected in the breath and sputum volatilome of individuals infected with PA (16, 17, 23, 24), as well as under specific bacterial culture conditions (25, 26). Consequently, it has been suggested as a potential non-invasive diagnostic biomarker for PA colonization. Methyl thiocyanate has emerged as an additional biomarker, exhibiting concentrations ranging from 2 to 21 ppbv in the exhaled breath of CF patients infected with PA, as well as in the bacterial culture headspace (18). Notably, the observed parallel correlation between HCN levels and methyl thiocyanate suggests that the synthesis of methyl thiocyanate by PA strains is contingent upon the production of HCN (18). Another VOC found in the breath of CF patients (13) and in the headspace of bacterial cultures (22, 27–32), is 2-aminoacetophenone (2-AA). This molecule, which imparts a distinctive ‘grape-like’ fruity odor on PA cultures, is known to modulate the virulence of PA by promoting a shift toward a chronic infection phenotype in lungs (33). Methyl ketones, such as 2-nonanone and 2-undecanone (30, 34) are likewise released by PA cultures *in vitro.* 2-nonanone, in particular, can be detected *in vitro* in bacterial cultures (30, 34–37) and *in vivo* as a marker for the detection of PA in the breath of bronchiectasis and CF septum samples (14).This detection sensitivity can be further enhanced by 19% when 2-nonanone is combined with 17 other detected VOCs in a sputum library (14). Other VOCs associated with PA under *in vitro* and *in vivo* conditions include hydrocarbons (e.g., 1-undecene (20, 22, 30, 32, 34, 35), 1-dodecene (35)), ketones (e.g., acetone (22, 27, 31, 37–39)), aldehydes (e.g., 3-methyl-1-butanol (14, 30, 34–36, 38)), acids (e.g., acetic acid (22, 27, 39)), alcohols (e.g., ethanol (27, 28, 31, 35, 38, 39), 1-butanol (34)), sulfur compounds (e.g., dimethyl sulfide (22, 32, 35, 37, 39), dimethyl disulfide (22, 30, 35, 37, 39), dimethyl trisulfide (30, 35, 37)), terpenes (e.g., 1-α-pinene (20), terpinen-4-ol (20)), and other compounds (e.g., 2,2,6-trimethyl-octane (20), indole (22, 27)). The identification of overlapping biomarkers among corroborating reports provides considerable encouragement that these VOCs are potentially PA-specific. A comprehensive list of core VOCs derived from PA has been compiled in **Tables 1**, **2**, incorporating both *in vitro* and *in vivo* published literatures, with associated diseases listed alongside.

### 2.2 Discrepancies and confounding factors between *in vitro* and *in vivo* findings on PA volatilome profiles

Collectively, the above studies suggest that PA-related VOC profiles may serve as sensitive and specific biomarkers for its identification and detection in human specimens (*in/ex vivo*), as well as in pure and mixed bacterial cultures. Despite these advances, integrating these biomarkers into the clinical diagnosis of PA lung infections remains challenging due to multiple confounding factors including differences in culture conditions, bacterial strains and phenotypes, host factors, the non-specific origins of many VOCs, and discrepancies between *in vitro* and *in vivo* research findings. Thus far, comprehensive translational research bridging *in vitro* and *in vivo* studies in human patients—an essential step for biomarker validation—remains limited. In 2013, Zhu et al. made the first attempt at comparing the *in vivo* and *in vitro* volatile profiles from the same PA and *Staphylococcus aureus* strains using a murine infection model (40). They showed a low similarity (25-34%) between VOC profiles of PA and *S. aureus* cultures *in vitro* to *in vivo* (40). Nevertheless, the VOC profiles were able to differentiate between mice with and without infection, between mice infected by PA versus S. aureus, and infection by different PA strains. In addition, the host immune response has a significant impact on the VOC profile. Bean et al., who reported the presence of unique breath prints including host-derived volatiles of inflammation that allow discrimination between healthy, active PA infection, and convalescent state (41). Furthermore, Fenn et al found that PA emitted fewer pathogen-specific VOCs when co-cultured with alveolar A549 human epithelial cells as compared to when PA was grown alone (42). All together, these findings suggest that VOC biomarkers are modulated by the availability of host environment, an essential consideration for understanding their biochemical origins.

Previous studies (29, 43) have also demonstrated how the bacterial culture environment (e.g., pH, CO_2_/O_2_ ratio, nutrient availability, and medium composition) influences the observed VOC profiles, highlighting PA’s ability to produce diverse VOCs while also posing a challenge in establishing a consensus panel of biomarkers for reliable *in vivo* detection. Moreover, it’s important to note that the VOC profile of PA can shift longitudinally, correlating with the adaptation of infection phenotypes (early vs. chronic), thus indicating the diagnostic potential for monitoring chronic CF lung infections through breath analysis (36). Overall, various confounding factors, including PA strains (31), bacterial culture media (29, 43), growth stage (biofilm vs. planktonic) (44), bacterial phenotypes (mucoid vs. non-mucoid) (45), and individual patient’s factors such as the stage of infection (36), diet (13, 46), and smoking (35), have all been shown to influence the composition of volatilome of PA.

### 2.3 Recent advances and concepts in PA volatilome profiling

As discussed above, many *in vitro* studies aimed at identifying distinct PA VOC biomarkers have not successfully translated into *in vivo* contexts for the identification of analogous volatilomes in clinical patients. The variability in VOC species observed in different studies, as outlined in **Tables 1**, **2**, raises the question of whether a single VOC is indicative of PA presence or if a distinct “pattern” of collective VOCs is, in fact, more reflective of this pathogen. Due to the limited success in developing clinical diagnostics based on selected *in vitro* volatile biomarkers, several techniques are now being explored to capture more comprehensive bacterial volatilomes for diagnostic purposes. Volatile profiling, also known as fingerprinting, is being explored through the application of chemical sensors along with gas chromatography (GC) and mass spectrometry (MS) techniques (12, 47–50). Since then, there has been notable success in utilizing the entire volatilome fingerprint for PA detection in both human (51) and murine models (40). The literature on this topic converges on the fact that volatile metabolites are related to infection pathogenesis as a whole, which may include both physiological and host response factors. Hence, it is generally a “pattern” of VOCs that signifies the presence of PA in clinical specimens, rather than the detection of an individual compound. The combination of multiple GC or GC-MS breath biomarkers, along with the use of the entire volatilome fingerprint, has proven to be a reliable strategy for diagnosing PA lung infections (12, 14, 52–54). Advances in analysis-methods and particularly in small and VOC-specific sensor-arrays resulted in cost-effective, miniaturized ‘eNose’ sensors. These devices, among other possible applications, have been used in pilot clinical studies to detect bacterial colonization in CF patients with bronchiectasis (55, 56), representing non-invasive diagnostic and monitoring tools for PA lung infections.

## 3 PA-derived secondary metabolites

In addition to the aforementioned VOCs, PA produces numerous important exoproducts and secondary metabolites that play a role in its pathogenicity and in the persistence of PA in the lung. These comprise the redox-active tricyclic phenazines, the quorum sensing (QS) molecules, siderophores, and exopolysaccharides that all have essential functions in the modulation of host cell behaviors. Some of the essential metabolites are listed below:

### 3.1 Phenazines

Phenazines represent a substantial category of nitrogen-containing heterocyclic compounds, which include the redox-active pyocyanin (PYO), phenazine-1-carboxylic acid (PCA), phenazine-1-carboxamide (PCN), 1-hydroxyphenazine, and 5-methylphenazine-1-carboxylic acid betaine (57, 58). These compounds are recognized as critical virulence factors of PA, playing significant roles in quorum sensing, biofilm formation, virulence expression, iron acquisition, oxidative stress, competition against other microbes within the same niche, and modulation of host responses (58–60). Through these multifaceted activities, phenazines greatly enhance the pathogenic potential and ecological adaptability of PA. Their detection in clinical specimens correlates with heightened virulence and adverse patient outcomes, particularly in cases of CF (61, 62).

### 3.2 Quorum sensing molecules

The QS systems in PA are a hierarchical network that orchestrates virulence factor expression and biofilm formation. This regulation is mediated by a variety of signaling molecules, including N-3-oxo-dodecanoyl homoserine lactone (3-oxo-C12-HSL), N-butanoyl-L-homoserine lactone (C4-HSL), *Pseudomonas* quinolone signal (PQS), 2-heptyl-4-hydroxyquinoline (HHQ), 2-(2-hydroxyphenyl)-thiazole-4-carbaldehyde (IQS), and 2-heptyl-4-hydroxyquinoline N-oxide (HQNO). Two acyl-homoserine lactone (AHL) QS systems, the Las and Rhl, are closely connected, and are involved in the synthesis of a variety of virulence factors such elastases, alkaline protease, rhamnolipids, phenazines, lectins, superoxide dismutase, and biofilm formation (63). The more recently identified PQS and IQS systems contribute additional layers of complexity to PA’s QS network (64). Notably, PQS, along with its precursor HHQ and the derivative HQNO, is frequently found in the sputum, bronchoalveolar fluid, and mucopurulent secretions of people with CF (65). In brief, QS systems allow PA to modulate gene expression in response to cell density, thus controlling important functions such as virulence, antibiotic resistance, and biofilm formation (64, 66). This intricate communication network significantly enhances the adaptability and pathogenic potential of PA in diverse environments.

### 3.3 Siderophores

The siderophores pyoverdine and pyochelin chelate iron from the host microenvironments and lysed RBCs. This system is not only essential for bacteria survival but also enhances pathogenicity during lung infection processes (67, 68).

### 3.4 Exopolysaccharides

Extracellular polysaccharides provide a barrier protecting bacteria against environmental factors, such as dehydration, bacteriophages and the host immune factors. PA synthesizes three main polysaccharides, including alginate, PSL, and PEL, all of which are important components of *in vitro* biofilms (69). The production of alginate is particularly noteworthy, as it imparts the mucoid phenotype of clinical PA isolates from CF lungs (70). These polysaccharides are important for the establishment of PA biofilms, providing a shield against host defenses such as reactive oxygen species (ROS) and phagocytosis (71–73), as well as enhancing resistance to antibiotics (74–76).

## 4 Effects of PA-derived metabolites on respiratory epithelial cells

**Figure 1:**
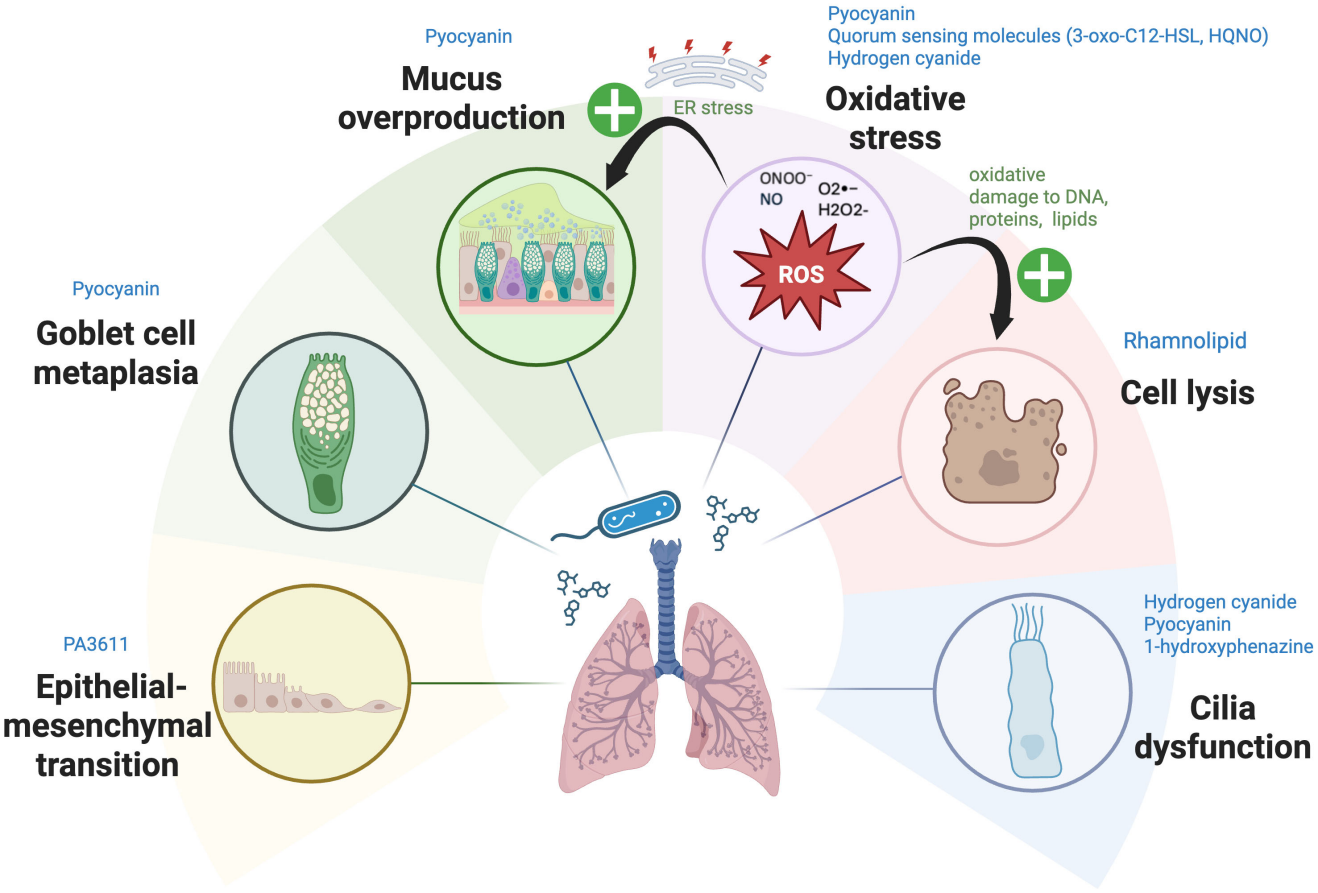
The mechanisms by which *Pseudomonas aeruginosa*-derived secondary metabolites affect respiratory epithelial cells. *Pseudomonas aeruginosa* (PA) employs multiple mechanisms to disrupt respiratory epithelial cells. Metabolites such as pyocyanin (PYO), hydrogen cyanide (HCN), and quorum-sensing (QS) molecules (3-oxo-C12-HSL, HQNO) generate reactive oxygen and nitrogen species (ROS, RNS), including superoxide (O2•−), hydrogen peroxide (H2O2), peroxynitrite (ONOO−), and nitric oxide (NO). These ROS and RNS disrupt mitochondrial electron transport, causing oxidative stress and damage to DNA, proteins, and lipids. Furthermore, excessive ROS and RNS generated by PA metabolites—such as PYO, 3-oxo-C12-HSL, HQNO, and HCN—perturb the respiratory epithelial barrier via activation of apoptosis pathways in epithelial cells and induce excessive mucus production by inducing endoplasmic reticulum (ER) stress. This stress further exacerbates mucus production and contributes to chronic inflammatory conditions. Rhamnolipid induce direct cell lysis, while HCN, PYO, and 1-hydroxyphenazine impair ciliary function, with PYO also driving mucus overproduction and promoting goblet cell metaplasia. Furthermore, PA3611, a quorum-sensing-regulated protein expressed by PA during infection, promotes epithelial-mesenchymal transition (EMT) in bronchial epithelial cells—a tissue remodeling process wherein epithelial cells lose their characteristics and differentiate into myofibroblasts. Image created with BioRender.com. Kuo, S. (2025) https://BioRender.com/f5fss63

The respiratory epithelium of human lung is the body’s first line of defense against inhaled germs, allergens, and pollutants, and plays a crucial role in the initiation of immune responses. Its primary innate immune functions encompass: (i) the production of mucus to ensnare pathogens; (ii) the expulsion of inhaled bacteria via ATP-dependent, coordinated mucociliary escalator; (iii) the release of antibacterial peptides and ROS; (iv) the initiation of wound healing processes after epithelial damage; and (v) the secretion of cytokines and chemokines to signal the immune system (77). The structural integrity of the epithelium, coupled with mucociliary clearance, pollutant metabolism, and production of antimicrobial and immune mediators, is essential for protecting the gas exchange units (alveoli) and submucosal layers from environmental inhalants (78). The integrity and function of respiratory epithelial cells are hence crucial for maintaining airway homeostasis. PA-derived metabolites and VOCs can disrupt airway epithelial functions in several ways summarized below (**Figure 1**).

### 4.1 Cytotoxicity via oxidative stress and direct cell lysis

PA metabolites can damage cellular and mitochondrial components, leading to cell death or dysfunction by generating excessive ROS and causing oxidative stress. These ROS interfere with multiple cellular functions in host cells, including electron transport, cellular respiration, and energy metabolism (60). PYO, a redox-active pigment and major virulence factor produced by PA, plays a significant role in oxidative stress generation by elevating intracellular levels of ROS, particularly superoxide (O_2_•−) and hydrogen peroxide (H_2_O_2_) via consumption of catalase-associated NADPH (60). These ROS cause oxidative damage to DNA, proteins, and lipids, thereby inhibiting key cellular enzymes and disrupting normal cellular functions (79, 80). Similarly, QS molecules such as 3-oxo-C12-HSL (81) and HQNO (82), along with the VOC HCN (24, 83, 84), disrupt electron transport in mitochondria, attenuating cellular respiration and inducing the generation of ROS. This, in turn, triggers apoptotic pathways in epithelial cells and compromises the integrity of the epithelial barrier (81, 85). Furthermore, rhamnolipids degrade lung surfactant and disrupt tight junctions, causing direct injury to tracheal and lung epithelial barrier (86).

### 4.2 Ciliary dysfunction

Phenazines and HCN, have detrimental effects on mucociliary clearance by directly impairing ciliary function. PYO and 1-hydroxyphenazine reduce ciliary beat frequency in the lungs, weakening the cilia’s ability to clear mucus and trapped particles from the airways (87). Likewise, HCN produced by PA, which is also a principal ‘ciliatoxic’ component found in cigarette smoke, significantly damages the cilia, disrupting their synchronized beating and hindering the efficient escalator movement of mucus that clears entrapped particles out of the respiratory system (88).

### 4.3 Goblet cell hyperplasia and mucus hypersecretion

**Figure 2:**
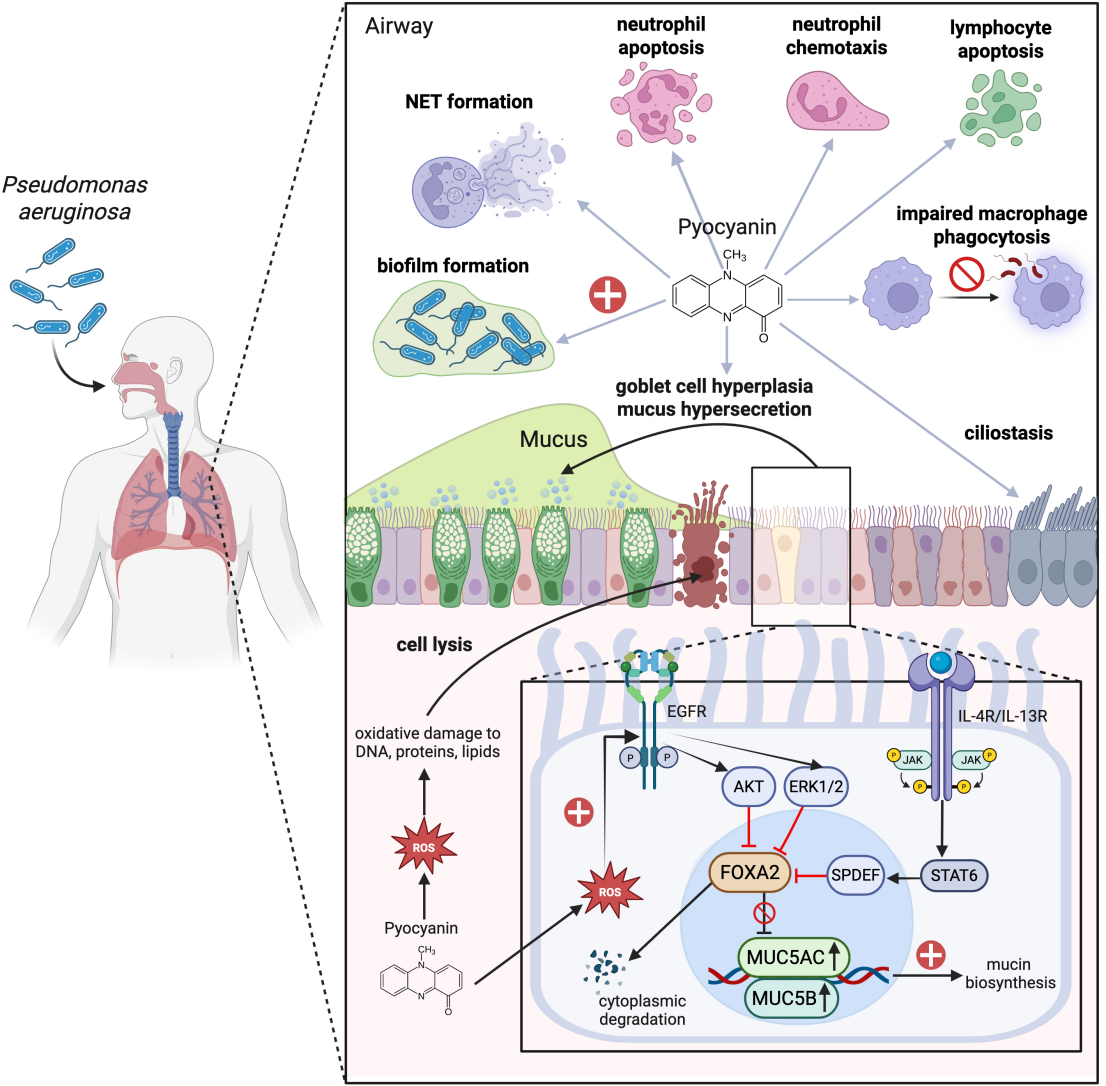
Mechanisms of respiratory impact and immune modulation by pyocyanin during *Pseudomonas aeruginosa* (PA) infection. Pyocyanin (PYO), a chemical redox pigment and the major virulence factor in PA, generates an oxidative burst through the increased production of intracellular reactive oxygen species (ROS) that damage DNA, protein, and phospholipids. These damages initiate apoptotic cascades and disruption of the respiratory barrier. Also, PYO reduces ciliary beats frequency, which has a detrimental effect on mucociliary clearance. Moreover, PYO causes goblet cell hyperplasia and mucus hypersecretion by suppressing FOXA2, a master regulator of mucus homeostasis, through activation of the EGFR-PI3K-AKT, EGFR-MEK-ERK, and IL-4/IL-13R-JAK-STAT6-SPDEF pathways. The ROS generated by PYO is additionally responsible for the promotion of chemokine and growth factor release which augment EGFR-induced mucin hyperproduction. In addition to the above effects, PYO also modulates both pro-inflammatory and anti-inflammatory immune responses. On the one hand, it increases neutrophil chemotaxis, and, on the other hand, it inhibits macrophage phagocytosis and activates the apoptosis of neutrophils, T lymphocytes, and B lymphocytes. Neutrophils drawn to tissue following chemotaxis exacerbate tissue damage via the release of ROS, proteases, and pro-inflammatory cytokines. PYO also induces the release of extracellular DNA with neutrophil extracellular traps (NETs) formation, which contribute to biofilm formation and persistent infection of PA in tissue. Image created with BioRender.com. Kuo, S. (2025) https://BioRender.com/5y1ycg1.

PYO plays a significant and multifaceted role in enhancing mucus hypersecretion and goblet cell metaplasia and hyperplasia during infections. PYO inactivates FOXA2, a transcriptional regulator of airway mucus homeostasis which ordinarily inhibits excessive goblet cell hyperplasia and metaplasia and mucus production (89, 90). Additionally, the ROS and reactive nitrogen species (RNS) generated by PYO also cause post-translational modifications of FOXA2, including nitrosylation, acetylation, and ubiquitination, which impair its capacity to bind to the promoter of the *MUC5B* gene (91). Subsequent investigations utilizing normal and CF and COPD-diseased primary and immortalized human airway cells, along with studies in mice, reveal that PYO inhibits FOXA2 expression via the activation of antagonistic signaling cascades, among others, EGFR-PI3K-AKT, EGFR-MEK-ERK, and IL-13R-STAT6-SPDEF, leading to goblet cell hyperplasia and metaplasia and overexpression and hypersecretion of mucus (89, 90, 92). Moreover, the ROS associated with PYO stimulate the release of inflammatory cytokines and growth factors that promote EGFR-dependent mucin secretion in airway epithelial cells (60, 93). Long-term chronic exposure (12 weeks) to PYO in murine airways results in goblet cell hyperplasia, airway fibrosis, destruction of alveolar spaces, and a shift towards a Th2 immune response marked by increased levels of Th2 cytokines IL-4 and IL-13. These cytokines further activate the STAT6 signaling pathway, exacerbating goblet cell hyperplasia and promoting excessive mucus production (92). Besides, PYO has been found to upregulate expression of sialyl-Lewis(x), a sugar modification of airway mucins to which PA preferentially adheres, utilizing this as part of its strategy to condition the airway for chronic infection (94). Consequently, a sophisticated interplay of autocrine and paracrine signaling pathways facilitates the mucin secretion induced by PYO in respiratory epithelial cells (**Figure 2**). Additionally, prolonged oxidative stress leads to an accumulation of improperly folded proteins within the endoplasmic reticulum (ER), resulting in ‘ER stress’ and the subsequent activation of the ‘unfolded protein response’ (UPR). This mechanism can further exacerbate mucus production and contribute to chronic inflammatory conditions (95–98) characterized by the secretion of proinflammatory cytokines. This release further escalates ER stress, creating a feedback loop that amplifies the inflammatory response (95, 99). Also, ER stress has been implicated in the initiation and progression of pulmonary fibrosis, with growing evidence suggesting that it also plays a role in obstructive lung diseases, pulmonary infections associated with CF, and lung cancer (100).

### 4.4 Epithelial-mesenchymal transition

As aforementioned, PA infects chronically diseased lungs (1). Epithelial injury triggers a sustained immune response, leading to emphysema and airway remodeling, which involves peribronchial fibrosis and possibly increased airway smooth muscle mass (101, 102). Pulmonary fibrosis develops as a complication of repeated PA infections, epithelial damage, and tissue repair. The EMT in bronchial epithelial cells—a tissue remodeling process where epithelial cells lose their characteristics and differentiate into myofibroblasts—plays a pivotal role in the progression of bronchial and pulmonary fibrosis and obliterative bronchiolitis (OB). These changes in cell proportions can result in goblet cell metaplasia/hyperplasia and increase mucus production, a hallmark of chronic bronchitis in COPD (103). Prolonged exposure to PYO has been shown to induce peribronchial fibrosis (92). PA3611, a putative QS-regulated protein produced by PA during infection (104), has been shown to promote EMT by integrin αvβ6-mediated activation of the TGF-β1-induced p38/NF-κB pathway, which causes mesenchymal markers to be upregulated and epithelial markers to be downregulated (105). In line with this, Borthwick et al. demonstrated that PA-activated monocytic cells can enhance TGF-β1-driven EMT in primary bronchial epithelial cells (106). These observations shed light on the association between PA infection and the increased likelihood of developing obliterative bronchiolitis following lung transplantation.

Overall, PA exerts its pathogenic effects through a multifaceted approach, including the production of ROS, inhibition of mucociliary blanket, and induction of goblet cell hyperplasia and metaplasia, mucus hypersecretion, and the promotion of EMT. A brief overview of documented PA-derived metabolites and VOCs, along with their implicated roles in respiratory epithelial pathology, is summarized in **Table 3**.

**Table 3:** Overview of PA-derived metabolites and VOCs in respiratory epithelial pathology.

PA-derived metabolites	Functions in respiratory pathology	References
Pyocyanin (PYO)	Generation of ROS	Gloyne et al. (2011) (79) Schwarzer et al. (2008) (80)
	Reduction in ciliary beat frequency	Wilson et al. (1987) (87)
	Goblet cell hyperplasia, metaplasia, and mucus hypersecretion	Rada et al. (2013) (60) Hao et al. (2012) (89) Choi et al. (2020) (90) Hao et al. (2013) (91) Caldwell et al. (2009) (92) Rada et al. (2011) (93) Jeffries et al. (2016) (94)
2-heptyl-4-hydroxyquinoline N-oxide (HQNO)	Generation of ROS	Rieger et al. (2020) (82)
Hydrogen cyanide (HCN)	Generation of ROS and interference with tissue oxygenation	Ryall et al. (2008) (24) Castric et al. (1975) (83) da Cruz Nizer et al. (2023) (84)
	Ciliary damage	Nair et al. (2014) (88)
N-3-oxo-dodecanoyl homoserine lactone (3-oxo-C12-HSL)	Induction of mitochondrial DNA oxidative injury	Maurice et al. (2019) (81)
Rhamnolipids	Degradation of lung surfactant and disruption of tight junctions	Zulianello et al. (2006) (86)
1-hydroxyphenazine	Reduction in ciliary beat frequency	Wilson et al. (1987) (87)
PA3611	Promotion of epithelial-mesenchymal transition	Shu et al. (2022) (105)

## 5 Influence of PA-derived metabolites on lung inflammation

Chronic inflammation is interconnected with mucus dysregulation and has a bidirectional relationship, that is, each of the two events serves as a cause for the other. Thus, PA-derived metabolites exacerbate pulmonary inflammation and disease courses through multiple mechanisms. It was noted that from the point of their role in lung inflammation, several metabolites have a significant influence on respiratory health and disease, as will be further discussed in the following section. Interestingly, these metabolites often exert a dual role in modulating inflammation during PA infection, promoting neutrophil chemotaxis while concurrently impairing host defense mechanisms. A comparison of PA-derived metabolites and VOCs involved in immune modulation during infection is presented in **Table 4**.

**Table 4:** Comparison of PA-derived metabolites and VOCs in immune modulation during infection.

Metabolites and VOCs	Functions in immune upregulation	References	Functions in immune downregulation	References
Pyocyanin (PYO)	Increases IL-8 expression in airway epithelial cells	Denning et al. (1998) (107) Pan et al. (2006) (108)	Induces neutrophil apoptosis	Allen et al. (2005) (115) Usher et al. (2002) (116) Managò et al. (2015) (117)
	Increases IL-8 secretion in U937 human monocytes	Chai et al. (2014) (109) Chai et al. (2013) (110)	Promotes neutrophil extracellular trap (NET) formation	Rada et al. (2013) (118)
			Inhibits T and B lymphocyte proliferation and induces lymphocyte apoptosis	Ulmer et al. (1990) (121) Nutman et al. (1987) (122) Mühlradt et al. (1986) (123) Oleiwi et al. (2015) (126)
			Impairs macrophage phagocytosis of apoptotic cells	Bianchi et al. (2008) (129)
			Inhibits phagocytosis-induced ROS release and reduces nitric oxide production in macrophages exposed to PA lipopolysaccharide (LPS)	Shellito et al. (1992) (127) Marreiro de Sales-Neto et al. (2019) (128)
1-hydroxyphenazine	Exerts proinflammatory effects on neutrophils, intensifying neutrophil-mediated tissue damage	Ras et al. (1990) (114)	Inhibits LPS-induced inflammation in RAW264.7 murine macrophages	Xiao et al. (2021) (131)
Phenazine-1-carboxylic acid (PCA)	Induces expression of IL-8 and ICAM-1	Denning et al. (2003) (132)	Reduces expression of RANTES and MCP-1	Denning et al. (2003) (132)
			Promotes biofilm formation	Wang et al. (2011) (133)
N-3-oxo-dodecanoyl homoserine lactone (3-oxo-C12-HSL)	Blocks anti-inflammatory PPARγ signaling in murine fibroblasts and human lung epithelial cells	Jahoor et al. (2008) (134) Cooley et al. (2010) (135)	Attenuates LPS-induced inflammation in RAW264.7 murine macrophages	Zhang et al. (2014) (137)
	Induces COX-2 expression and PGE2 production in lung fibroblasts	Smith et al. (2002) (136)	Promotes apoptosis in macrophages, neutrophils, lymphocytes, and platelets	Tateda et al. (2003) (138) Kushwaha et al. (2023) (139) Yadav et al. (2021) (140)
2-undecanone	Activates neutrophils via the Gαi–phospholipase C signaling pathway	Jeong et al. (2022) (148)	Induces neutrophils apoptosis	Jeong et al. (2022) (148)
2-aminoacetophenone (2-AA)			Suppresses pro-inflammatory cytokine expression in THP-1 human monocytes and RAW264.7 murine macrophages	Bandyopadhaya et al. (2012) (143) Bandyopadhaya et al. (2016) (144) Bandyopadhaya et al. (2017) (145)
			Inhibits autophagy and lipid synthesis in RAW264.7 murine macrophages	Chakraborty et al. (2023) (146)

### 5.1 Pyocyanin

PYO, in particular, plays a complex role in modulating inflammation during PA infection. First, PYO has several mechanisms that promote inflammation, and is known to increase the expression of interleukin-8 (IL-8) in airway epithelial cells that involve oxidative stress and kinase signaling pathways (107, 108). Additionally, it acts in synergy with pro-inflammatory cytokines such as TNF-α and IL-1α resulting in an amplified production of IL-8 (107). Chai et al. conducted further studies that indicated PYO significantly increases IL-8 secretion in U937 cells, a human monocyte cell line, in a time- and concentration-dependent fashion. Their research suggests that this effect is mediated through the activation of specific signaling pathways, including protein kinase C (PKC), p38, and ERK mitogen-activated protein kinases (MAPKs), in addition to nuclear factor-kappa B (NF-κB) (109, 110). The antioxidant N-acetyl cysteine was found to effectively inhibit the expression of IL-8, suggesting a ROS-dependent mechanism (109). As a potent neutrophil chemoattractant, elevated IL-8 levels play a crucial role in driving the pronounced neutrophil infiltration frequently observed in PA infections. Neutrophils are central to the pathogenesis of CF and other respiratory disorders, where their elevated presence in lung tissue often intensifies the inflammatory response (111). Their accumulation, while aimed at clearing bacterial infections, inadvertently contributes to lung damage through the release of proteases, ROS, and pro-inflammatory cytokines, which can harm the surrounding tissues and exacerbate disease progression (112). In particular, neutrophils release neutrophil elastase, myeloperoxidase and H_2_O_2_, which are key components of the peroxidase system and potent contributors to oxidative stress (113). This oxidative stress, in turn, amplifies cellular damage and further escalates inflammatory responses in the lungs (114).

While PYO possesses pro-inflammatory properties, it is also able to inhibit various arms of the immune responses in neutrophils, lymphocytes, and macrophages. Even as it is extremely neutrophilic, PYO can induce neutrophil apoptosis, thereby hampering their host defense mechanisms and allowing PA to evade immune clearance (115, 116) through stimulation of mitochondrial ROS release and activation of mitochondrial acid sphingomyelinase (117). Moreover, PYO induces extracellular DNA (eDNA) and neutrophil extracellular traps (NETs) release in a dose-dependent manner, a process that requires NADPH oxidase and involves c-Jun N-terminal kinase (JNK) and phosphatidylinositol 3-kinase (PI3K) pathways (118). NETs in their turn escalate biofilm formation (119) the latter is a well-established driver of persistent infections that are difficult to eradicate (120). Besides, PYO exhibits dose-dependent effects on B and T lymphocyte function (121). PYO inhibits T lymphocyte proliferation by blocking the release of IL-2 and reducing IL-2 receptor expression on T cells (121–123). This inhibition reduces immunoglobulin secretion by B lymphocytes and decreases lymphocyte proliferation, ultimately leading to a diminished immune response against PA (123–125). Of note, it was shown that toxic effects on T and B lymphocyte proliferation could be induced by PYO concentrations as low as 0.5 µg/mL (121). This is further supported by subsequent study showing that PYO induces DNA fragmentation in human peripheral blood lymphocytes, leading to their apoptosis (126). PYO suppresses phagocytosis-induced ROS generation and subsequently decreases the production of nitric oxide in macrophages upon the treatment of PA’s lipopolysaccharides (LPS) (127). These results were further supported by an independent study showing that PYO exerts anti-inflammatory effects by downregulating the production of nitric oxide, TNF-α, and IL-1β in LPS-activated murine macrophages (128). Additionally, macrophage phagocytosis of apoptotic cells was also impaired by the presence of PYO, which was related to the generation of intracellular ROS and alterations in small GTPase signaling (129). These multiple effects of PYO on immune cells contribute to PA’s ability to evade host defenses and establish chronic infections, particularly in immunocompromised individuals (130). The important roles of PYO in infection and pulmonary inflammation are also summarized in **Figure 2**.

### 5.2 1-hydroxyphenazine and phenazine-1-carboxylic acid

Other phenazine compounds, such as 1-hydroxyphenazine and PCA, have also been reported to interfere with the host mucosal immune responses. Similar to PYO, 1-hydroxyphenazine has proinflammatory effects on neutrophils that may intensify neutrophil-mediated tissue damage during infection (114). Intriguingly, 1-hydroxyphenazine was later characterized as having anti-inflammatory activity toward murine macrophages, inhibiting LPS-induced inflammation in RAW264.7 cells *in vitro* (131). PCA has been found to induce expression of both IL-8 and ICAM-1, but simultaneously reduces the expression of RANTES and monocyte chemoattractant protein-1 (MCP-1) (132). In human airway epithelial cells, PCA is also linked to heightened intracellular oxidant generation (132). These activities are further inhibited by antioxidants, suggesting that oxidative stress is integral to these mechanisms (132). Furthermore, PCA is implicated in promoting bacterial biofilm formation through the acquisition of ferrous iron in the later stages of infection (133).

### 5.3 N-3-oxo-dodecanoyl homoserine lactone

The PA QS signaling molecule 3-oxo-C12-HSL can also modulate the function of a variety of mammalian cell types, including lymphocytes, macrophages, neutrophils, platelets, fibroblasts, and respiratory epithelial cells. By acting as an agonist of PPARβ/δ and antagonist of PPARγ, 3-oxo-C12-HSL induces proinflammatory responses in host cells by blocking anti-inflammatory PPARγ signaling in murine fibroblasts and human lung epithelial cells (134, 135). It also stimulates the formation of cyclooxygenase-2 and prostaglandin E2 production in lung fibroblasts, hence contributing to inflammation and lung pathology (136). Conversely, 3-oxo-C12-HSL attenuates LPS-induced inflammation in RAW264.7 mouse macrophage cell by activating the unfolded protein response, which suppresses NF-κB activation (137). 3-oxo-C12-HSL particularly facilitates the induction of apoptosis in diverse immune cells, including macrophages (138), neutrophils (138), lymphocytes (139), and platelets (140). These studies further reinforce the concept that QS AHLs not only regulate bacterial virulence but also modulates various cellular functions that are essential for host inflammation and immune defenses.

### 5.4 2-aminoacetophenone

The PA VOC, 2-AA, plays a complex role in inflammation and infection. 2-AA silences acute virulence functions while promoting chronic infection phenotypes in PA by modulating the virulence regulator MvfR and inducing biostability (33). Although not demonstrated in lungs, 2-AA has been shown to trigger mitochondrial dysfunction in skeletal muscle, reducing the rate of ATP synthesis and compromises muscle function (141, 142). A decline in energy production, coupled with mitochondrial dysfunction, may create conditions that favor infections and contribute to host tolerance of pathogens, promoting their persistence—an important step in establishing chronic infections (33, 141). Additionally, 2-AA has been found to regulate HDAC1 activity and NF−KB interactions, suppressing pro-inflammatory cytokine expression in human monocytes THP-1 cells and mouse macrophage RAW264.7 cell (143–145). Recently, Chakraborty et al. found that 2−AA inhibits murine macrophage processes such as autophagy and lipid synthesis (146) and re-wiring cellular bioenergetics through the PGC−1/ERR axis, reducing bacterial clearance (146, 147). Moreover, in mouse models of PA infection, pretreatment with 2-AA yields a higher survival rate compared to control mice, even with increased bacterial burden (143). Collectively, these observations suggest that 2-AA has a multifunctional role in PA infection, regulating immunological and metabolic processes to promote host tolerance and bacterial persistence, promoting chronic infection.

### 5.5 2-undecanone

Another VOC 2-undecanone, which is produced by PA during infection, has recently been identified as a potent activator of neutrophils through the Gαi-phospholipase C pathway. However, this activation subsequently leads to a reduction in the bactericidal capabilities and promotes apoptosis of neutrophils, potentially aiding PA in escaping immune detection (148).

## 6 Conclusion

There is a burgeoning interest in microbial VOCs, with a growing number of research efforts focused on understanding their production and functional roles. In this review, we summarize the major species of PA-derived VOCs and discuss the potential and limitations of VOCs in the non-invasive diagnosis of chronic lung infections, calling for more intensified translational research to bridge *in vitro* and *in vivo* findings. Advances in analytical techniques are enabling increasingly broader VOC profiling, steering away from individual biomarkers and towards more comprehensive metabolic profiles that better represent PA infections in the clinical niche. PA-derived secondary metabolites, including VOCs, initiate a multifaceted array of signaling pathways and molecular events in airway epithelial cells, leading to epithelial and ciliary injury, mucostasis, EMT, and disturbed local immune responses. These mechanisms include the activation of oxidative stress pathways, ER stress, inflammatory signaling, mucin gene regulation, and more. In addition, the influence of PA metabolites on lung inflammation presents multifaceted interactions between pathogenicity and the host immune response. The phenazines PYO, 1-hydroxyphenazine, and PCA represent how PA-metabolites can worsen and moderate inflammatory processes in the various subpopulations of immune cells in lungs. PYO, through its twin role in triggering neutrophil infiltration and simultaneously inactivating their host defense functions, highlights the complexities of the inflammatory response to PA infection. Also, 3-oxo-C12-HSL as well as 2-AA are other metabolites that showcase the delicate connection between the host immune system and the QS communication of bacteria. This review also highlights PA-derived metabolites’ participation in chronic lung inflammation and development of the disease course. Deeper insights into these complex interactions and disease mechanisms opens avenues for targeting PA metabolites and virulence factors in therapeutic and diagnostic strategies, improving outcomes in PA infections.
